# Supplementary material for: Content-rich biological network constructed by mining PubMed abstracts
Source: BMC Bioinformatics. 2004 Oct 8;5:147. doi: 10.1186/1471-2105-5-147 (PMC528731; doi:10.1186/1471-2105-5-147)
Supplement: Additional File 2 — The original results of the above study (non-essential files are deleted to keep the file size under the limit set by BMC bioinformatics). [file 1471-2105-5-147-S2.bz2 › chilibotAdditionalFile2/dip50/100ID7759493E427/html/P_NP.html]

 


 **P** and **NP** 
  
Found 2734 abstracts in PubMed, retrieved 50.  
 

 What does Google say? 
 PDF only 
| .edu only 

---

**Interactive relationship** (e.g. stimulation, inhibition, etc)

**Neutral relationship**- Liver protein concentration was less  **P**  = 0.07 in PR than in  **NP**  heifers  **NP**  = 291.1 vs. PR = 210.5 33.9 mg g.  Ref: 12854824 J Anim Sci, 2003
- HR baroreflex response DeltaHR DeltaMAP to PE LBNP  **NP**  was lower  **P**  = 0.015 after maximal exercise 0.43 0.15 beats.min 1.mm Hg 1 compared with the control condition 0.83 0.14 beats.min 1.mm Hg 1.  Ref: 12783045 Med Sci Sports Exerc< MedlineTA>Med Sci Sports Exerc, 2003
- However, the open probability was higher in  **NP**  cells compared to NT cells  **P**  <.  Ref: 12768737 Zhonghua Er Bi Yan Hou Ke Za Zhi, 2000
- The independent RFs of  **NP**  were a history of  **NP**  during the previous 6 months OR = 4.50 and oxygen therapy OR = 16.15,  **P**  < ;.  Ref: 12834521 J Am Geriatr Soc, 2003
- **P**  patients were weaned significantly faster than  **NP**  patients median, 9 days and 20 days, respectively.  Ref: 12813261 Pediatr Crit Care Med, 2000
- RESULTS The expression of VEGF mRNA was significantly higher in epithelial cells of  **NP**  than in IT exposed to pro inflammatory cytokines or hypoxia  **P**  <.  Ref: 12775240 Chin Med J Engl , 2003
- The plots of cellular SRA against the two insecticides and PNP  [ **NP** ]  were linear over the ranges of 0.5 2.5 mM for  **P** . putida C 11 and BA 11 and 0.5 1.0 mM for A. calcoaceticum A 122.  Ref: 12782463 Biosens Bioelectron, 2003
- Although the nucleoprotein  [ **NP** ]  was immunogenic on its own, mice immunized with the nucleoprotein  [ **NP** ]  plus wild type LT produced significantly high antibody responses  **p** <.  Ref: 12789125 Afr Health Sci, 2001
- 3  **NP**  treated rats receiving AAV GDNF displayed 70% more NeuN immunoreactive neurons compared to 3  **NP**  treated rats receiving AAV GFP  **P**  = 0.002.  Ref: 12781994 Exp Neurol, 2003
- **P**  rats were found to have significantly greater brain damage, compared with  **NP**  rats, in the posterior perirhinal and posterior entorhinal cortexes, 239% 50%  **p**  <.  Ref: 12878913 Alcohol Clin Exp Res, 2003
- **P**  patients requiring short term opiate use also weaned significantly faster than short term  **NP**  patients median, 5 days and 21.5 days, respectively.  Ref: 12813261 Pediatr Crit Care Med, 2000
- Phospho extracellular signal regulated kinase immunohistochemistry stained prominently in damaged brain areas.CONCLUSIONS The  **P**  rat line, a genetic model of alcoholism, shows greater region specific brain damage due to binge ethanol treatment than its genetic counterpart, the  **NP**  rat line.  Ref: 12878913 Alcohol Clin Exp Res, 2003
- Enzymatic transglycosylation using four possible monodeoxy analogs of  **p**  nitrophenyl alpha D glucopyranoside Glc alpha O pNP  [ **NP** ] , modified at the C 2, C 3, C 4, and C 6 positions 2D, 3D, 4D, and 6D Glc alpha O pNP  [ **NP** ] , respectively, as glycosyl donors and six equivalents of ethyl beta D thioglucopyranoside Glc beta S Et as a glycosyl acceptor, to yield the monodeoxy derivatives of glucooligosaccharides were done.  Ref: 12834279 Biosci Biotechnol Biochem, 2003
- The average exercise related decreases in UtBF  **P**  16 4% vs  **NP**  48 4% and UtC  **P**  27 4% vs  **NP**  54 4% were attenuated in the  **P**  rabbits.  Ref: 12869366 Am J Physiol Regul Integr Comp PhysiolAm J Physiol Regul Integr Comp Physiol, 2003
- ... it was reduced by 27%  **P** < ; 0.05 in the 3  **NP**  MPTP model and in animals treated with either 3  **NP**  27%,  **P** < ; 0.05 or MPTP 23%,  **P** < ; 0.05 alone.  Ref: 12764627 Acta Neuropathol Berl < MedlineTA>Acta Neuropathol Berl , 2003
- Further, loss of tyrosine hydroxylase positive neurons in substantia nigra pars compacta SNc was attenuated after 3  **NP**  MPTP treatment compared to that observed after MPTP 40% vs 74%,  **P** < ; 0.001 and MPTP 3NP treatment 55% vs 74%,  **P** < ; 0.01.  Ref: 12764627 Acta Neuropathol Berl < MedlineTA>Acta Neuropathol Berl , 2003
- Pregnant and  **NP**  ileal energy use increased linearly  **P**  <.  Ref: 12854824 J Anim Sci, 2003
- both PR and  **NP**  decreased with time, but  **NP**  increased on d 270  **P**  = 0.09.  Ref: 12854824 J Anim Sci, 2003
- 0.01, the conductance of Na channels in  **NP**  cells was strongly increased by IL 1 beta 100 ng ml  **P**  <.  Ref: 12768737 Zhonghua Er Bi Yan Hou Ke Za Zhi, 2000

**Non-interactive relationship** (e.g. studied together, co-existance, homology, etc.)

- 2 Application of IL 1 beta increased significantly in the open probability of Na channels in NT and in  **NP**  cells  **P**  <.  Ref: 12768737 Zhonghua Er Bi Yan Hou Ke Za Zhi, 2000
- To this aim, rats of the sNP and Sardinian alcohol preferring sP lines were exposed to a 108 day sucrose fading procedure, which was virtually identical to that previously used with  **NP**  and  **P**  rats.  Ref: 12878272 Alcohol, 2003
- For producing a high level of recombinant mumps virus nucleoprotein  [ **NP** ]  the expression system of yeast  **P** . pastoris was employed.  Ref: 12770503 J Biotechnol, 2003
- BACKGROUND In a previous study, neuropeptide Y NPY administered into the lateral ventricles decreased ethanol intake in alcohol preferring  **P**  rats but not in alcohol nonpreferring  **NP**  or unselected Wistar rats.  Ref: 12824809 Alcohol Clin Exp Res< MedlineTA>Alcohol Clin Exp Res, 2003
- This work is also the first to provide molecular data on  **NP** ,  **P**  and L genes of typical pPMV 1.  Ref: 12803469 Virus Genes, 2003
- We examined the possibility of measuring the organophosphorus aromatic nitro insecticides metaphos and sumithion as well as their hydrolysis product  **p**  nitrophenol PNP  [ **NP** ]  by the specific respiratory activity SRA of Pseudomonas putida C 11,  **P** . putida BA 11, and Acinetobacter calcoaceticum A 122.  Ref: 12782463 Biosens Bioelectron, 2003
- Nine of ten  **NP**  and seven of ten  **P**  patients were on continuous fentanyl infusions, and the remainder were on continuous morphine infusions.  Ref: 12813261 Pediatr Crit Care Med, 2000
- Pregnancy did not affect the average steady state renal SNA response expressed relative to maximum activity  **P**  24 1% vs  **NP**  23 2% of maximum smoke elicited activity and increased the average renal SNA response expressed relative to resting activity  **P**  155 19% vs  **NP**  84 23% from rest,  **P** =0.03 At rest, UtBF  **P**  13 3 vs  **NP**  1.9 0.3 ml min and UtC  **P**  22 5 vs  **NP**  2.8 0.5 mmHg ml.  Ref: 12869366 Am J Physiol Regul Integr Comp PhysiolAm J Physiol Regul Integr Comp Physiol, 2000
- The uterine artery blood flow and renal sympathetic nerve activity SNA responses to treadmill exercise were evaluated in 12 nonpregnant  **NP**  and 17 term pregnant  **P**  rabbits.  Ref: 12869366 Am J Physiol Regul Integr Comp PhysiolAm J Physiol Regul Integr Comp Physiol, 2000
- The discussion focuses mainly on recent studies with genetic animal models including mutant mice lacking specific NPY receptor and selectively bred rodents, namely the Indiana alcohol preferring  **P**  and alcohol nonpreferring  **NP**  rats and the Indiana high alcohol drinking HAD and low alcohol drinking LAD rats.  Ref: 12818714 Physiol Behav, 2003
- Ces10 exhibited a marked preference for short chain fatty acids, yielding the highest activity with  **p**   **NP**  propionate optimal pH 7.4, optimal temperature 37 degrees C.  Ref: 12764567 Appl Microbiol Biotechnol, 2003
- Following 3  **NP** , the performance by AAV GFP treated rats on a raised platform motor task deteriorated while the performance by AAV GDNF treated rats was near normal  **P**  <.  Ref: 12781994 Exp Neurol, 2003
- Three avian Paramyxovirus type 1 aPMV 1 isolated from pigeons duringpigeon paramyxovirosis outbreaks were molecularly characterised by sequencing parts of the six genes  **NP** ,  **P** , M, F, HN and L of each strain.  Ref: 12803469 Virus Genes, 2003
- Paraplegic patients were more likely to have MS pain  **P** =0.001 and  **NP**  pain  **P** =0.046.  Ref: 12799608 Int J Rehabil Res, 2003
- snail, abalone, and limpet origins were investigated with assorted  **p**  nitrophenyl pNP  [ **NP** ]  di O sulfonated beta D galactopyranosides and beta lactosides SO 3 Gal, 6 SO 3 Lac, 6SO 3 Gal, 6 SO 3 Gal, 4 SO 3 Gal 5, and SO 3 GalNAc 6.  Ref: 12851934 Chembiochem, 2003
- Animals were killed, transcardially perfused, and fixed, and their brains were removed, sectioned, and stained by using the amino cupric silver stain of de Olmos or by using immunohistochemistry for phospho extracellular signal regulated kinases and other antigens.RESULTS Significant brain damage was found in the olfactory bulbs, posterior perirhinal cortex, and entorhinal cortex in both  **P**  and  **NP**  rats.  Ref: 12878913 Alcohol Clin Exp Res, 2003
- The GST activity then decreased in a time dependent manner in treatment groups, and this decrease was lower in the livers of fish treated with 66 and 220& ; mgr ; g  **NP**  L than in control fish after 3 weeks of exposure  **P** < ; 0.05.  Ref: 12804523 Environ Res< MedlineTA>Environ Res, 2003
- Parts per million and parts per billion concentrations of these species were found in the air passing through the tubing the plasticizers di 2 ethylhexyl phthalate DEHP and di ethyl phthalate DEP, the antioxidants butylated hydroxy toluene BHT and  **p**  nonylphenol  **p**   **NP** , and the contaminant from commercial preparation of DEHP 2 ethylhexanol 2 EH.  Ref: 12833390 Biomed Chromatogr, 2003
- Mature male medaka were continually exposed to four chemicals,  **p**  n nonylphenol  **p**  n  **NP** , nonylphenol  **p**   **NP** , bisphenol A BPA and 17beta estradiol E2 to evaluate their estrogenic activities in the laboratory.  Ref: 12830940 Water Sci Technol, 2003
- 0.01, but ileal energy use by PR was less throughout gestation  **P**  = 0.07 than ileal energy use by  **NP** .  Ref: 12854824 J Anim Sci, 2003
- To investigate the role of genetics in binge drinking induced brain damage, we studied bidirectionally selected rat lines, the alcohol preferring  **P**  and the alcohol nonpreferring  **NP**  rat lines.METHOD  **P**  and  **NP**  rats were treated with a 4 day binge ethanol protocol.  Ref: 12878913 Alcohol Clin Exp Res, 2003
- Although few systems have been studied in detail, F like T4SS appear to be involved in the transfer of DNA only whereas  **P**  and I type systems appear to transport protein or nucleoprotein  [ **NP** ]  complexes.  Ref: 12855161 FEMS Microbiol Lett, 2003
- The relative potencies of these chemicals descended in the order of E2>> **p**   **NP** >BPA.  Ref: 12830940 Water Sci Technol, 2003
- There was no difference between NT and  **NP**  cells in the incidence  **P**  > 0.05 and in the conductance  **P**  > 0.05.  Ref: 12768737 Zhonghua Er Bi Yan Hou Ke Za Zhi, 2000
- DeltaFENa was 7.22 1.03% for  **NP**  VE, 9.89 1.85% for  **NP**  L NMMA, and 17.66 1.85% for  **NP**  VE L NMMA  **P** < ; 0.05 vs.  **NP**  VE and  **NP**  L NMMA.  Ref: 12876067 Am J Physiol Renal PhysiolAm J Physiol Renal Physiol, 2000
- The yield of purified nucleocapsid  [ **NP** ]  like particles from  **P** . pastoris constituted 2.1 mg per 1 g of wet biomass and was considerably higher in comparison to the other expression systems.  Ref: 12770503 J Biotechnol, 2003
- Enzyme assays using  **p**  nitrophenyl esters  **p**   **NP**  esters with different acyl chain lengths as the substrate confirmed the anticipated esterase activity.  Ref: 12764567 Appl Microbiol Biotechnol, 2003
- Much greater vitellogenin compared to the background levels were induced in the male medaka by separate exposure to 100 microg L of  **p**   **NP** , 00 microg L of BPA and 0.05 microg L of E2.  Ref: 12830940 Water Sci Technol, 2003
- Furthermore, an increase in the activity of glutathione S transferase GST was found in the liver of fish exposed to 220& ; mgr ; g  **NP**  L for 1 week  **P** < ; 0.05.  Ref: 12804523 Environ Res< MedlineTA>Environ Res, 2003
- Although no chromogenic activity was detected from pNP  [ **NP** ]  glycosides, TLC analysis revealed the release of  **p**  nitrophenyl glycosides and cellodextrins from these substrates, suggesting that Cel48C acts from the reducing ends of the sugar chain.  Ref: 12823562 Eur J Biochem, 2003
- Significant methadone calculation discrepancy occurred in one  **NP**  patient but in no  **P**  patients.  Ref: 12813261 Pediatr Crit Care Med, 2000
- MEASUREMENTS AND MAIN RESULTS Patient age, gender, and diagnosis were similar in both nonprotocol  **NP**  and protocol  **P**  groups  **p**  = NS.  Ref: 12813261 Pediatr Crit Care Med, 2000
- Covalent attachment of 2C5 antibody was performed via a micelle incorporated PEG PE with the distal terminus of the PEG block activated with  **p**  nitrophenylcarbonyl group pNP  [ **NP** ]  PEG PE.  Ref: 12881194 J Drug Target, 2003
- Binge Ethanol Treatment Causes Greater Brain Damage in Alcohol Preferring  **P**  Rats Than in Alcohol Nonpreferring  **NP**  Rats.  Ref: 12878913 Alcohol Clin Exp Res, 2003
- Time dependent  **NP**  bioaccumulation was detected in the tissues of fish exposed to 220& ; mgr ; g  **NP**  L  **P** < ; 0.05 and histopathological changes were observed in the livers of fish exposed to 220& ; mgr ; g  **NP**  L.  Ref: 12804523 Environ Res< MedlineTA>Environ Res, 2003
- VEGF levels were higher in  **NP**  epithelial cells than those of IT  **P**  <.  Ref: 12775240 Chin Med J Engl , 2003
